# Short-term home-based multimodal prehabilitation for perioperative functional recovery in prefrail and frail older men undergoing radical prostatectomy: a randomized controlled trial

**DOI:** 10.1186/s12877-026-08031-3

**Published:** 2026-07-24

**Authors:** Zijuan Wang, Nan Wang, Bifang Xu, Jiaxin Qiu, Xianfeng Ye, Shenglan Lin, Li Ouyang, Shaoxin Feng, Na Li

**Affiliations:** 1 Shengli Clinical Medical College, Fujian Medical University, Fuzhou, China; 2 Fuzhou University Affiliated Provincial Hospital, Fuzhou, China; 3 The Affiliated Hospital of Qingdao University, Qingdao, China; 4 Department of Urology, Fujian Provincial Hospital, Fuzhou, China; 5 The School of Nursing, Fujian Medical University, Fuzhou, China; 6 Department of Gastroenterology, Fuzhou University Affiliated Provincial Hospital, Fuzhou, China; 7 Department of Nursing, Fujian Provincial Hospital, Fuzhou, China

**Keywords:** Prehabilitation, Frail, Radical prostatectomy, Functional capacity, Enhanced recovery, Older adults

## Abstract

**Background:**

Prefrailty and frailty are associated with reduced physiological reserve and heightened vulnerability to surgical stress, which may compromise early recovery after radical prostatectomy. Although multimodal prehabilitation may enhance surgical readiness, evidence remains limited for prefrail and frail older men undergoing radical prostatectomy within the short preoperative waiting period typical of routine practice.

**Methods:**

This prospective, outcome-assessor-blinded, parallel-group randomized controlled trial enrolled 80 men aged 60 or older with prostate cancer who were classified as prefrail or frail and scheduled for elective radical prostatectomy. Participants were randomly assigned to short-term home-based multimodal prehabilitation plus usual care or usual care alone. The intervention was delivered from enrolment until the day before surgery and included lifestyle-integrated exercise, nutritional optimisation, psychological support, pelvic floor muscle training, respiratory exercises, and perioperative health-behaviour guidance. The primary outcome was functional capacity measured by the 6-min walk test (6MWT) across the perioperative period. The primary comparison was the between-group difference in change in 6MWT from baseline to the day before surgery. Supportive secondary outcomes included frailty-related physical performance measures, and exploratory outcomes encompassed selected perioperative recovery indicators. Assessments occurred at baseline, the day before surgery, at discharge, and 4 weeks postoperatively.

**Results:**

All 80 randomized participants were included in the intention-to-treat analysis. The median duration of prehabilitation was 12 days. For the defined primary comparison, the prehabilitation group improved by 27.70 ± 16.86 m in 6MWT from baseline to the day before surgery, whereas the usual care group changed by − 3.12 ± 3.62 m, yielding a between-group difference of 30.82 m (95% CI, 25.32 to 36.33; *P <* 0.001). The generalized estimating equation (GEE) analysis showed a more favourable perioperative 6MWT trajectory in the prehabilitation group.

Frailty-related physical performance outcomes showed directionally consistent improvements. Exploratory perioperative outcomes showed signals of earlier ambulation, faster gastrointestinal recovery, and shorter postoperative hospital stay. The mean overall programme execution rate was 79.3%, and no intervention-related adverse events were reported.

**Conclusions:**

Short-term home-based multimodal prehabilitation improved the perioperative trajectory of functional capacity in prefrail and frail older men undergoing radical prostatectomy. Supportive and exploratory findings suggest potential benefits for early functional recovery; however, larger multicentre trials with longer follow-up are needed to confirm durability and broader clinical relevance.

**Trial registration:**

Chinese Clinical Trial Registry, ChiCTR2400094812. Registered on 27 December 2024. Retrospectively registered.

**Supplementary Information:**

The online version contains supplementary material available at https://doi.org/10.1186/s12877-026-08031-3.

## Background

Prostate cancer is the second most commonly diagnosed malignancy in men worldwide [1]. For localized or locally advanced disease, radical prostatectomy (RP) remains a standard curative treatment [2]. As the global population ages [3], a growing proportion of patients undergoing RP are older adults with diminished physiological reserve [4]. In this context, early postoperative recovery is increasingly limited by patients’ baseline functional capacity rather than solely by surgical technique [5]. Therefore, preserving and improving functional capacity across the perioperative period has become a clinically meaningful target in the care of older surgical patients.

Chronological age alone is an incomplete indicator of surgical vulnerability. Instead, prefrailty and frailty, characterised by impaired physiological reserve and diminished adaptive capacity to stressors, are increasingly recognised as more informative risk states [6, 7]. Accumulating evidence indicates that frailty is associated with delayed functional recovery, prolonged hospitalisation, and increased mortality [8–10]. Among men undergoing RP, emerging data further suggest that frailty is linked to worse early postoperative functional outcomes and a higher risk of major complications [11–13]. Importantly, frailty is not a fixed state and may be modifiable, especially at the prefrailty stage. These findings underscore the critical importance of identifying high-risk individuals preoperatively and implementing targeted strategies to enhance resilience to surgical stress.

Prehabilitation has been proposed as a strategy to improve surgical readiness by optimising physical, nutritional, and psychological status before surgery [14, 15]. Multimodal prehabilitation is increasingly recognized in oncologic surgery for its potential to enhance functional capacity and early postoperative recovery [16]. However, current evidence is largely derived from heterogeneous surgical populations and intervention protocols that typically require several weeks of preparation [17, 18]. This creates an important mismatch with routine radical prostatectomy pathways [19], in which the interval between surgical decision-making and operation may be short and variable [20, 21]. For prefrail and frail older men, the clinical question is therefore not only whether prehabilitation works under ideal conditions, but whether a brief, pragmatic, home-based programme can meaningfully influence functional capacity within the constrained preoperative waiting period.

Therefore, we conducted a randomized controlled trial to evaluate a short-term home-based multimodal prehabilitation programme delivered during the actual preoperative waiting period before radical prostatectomy. The primary aim was to determine its impact on the perioperative trajectory of functional capacity, measured by the 6-min walk test, in prefrail and frail older men. Frailty-related physical performance measures and selected perioperative recovery indicators were assessed to support interpretation of the primary functional outcome and to generate hypotheses for future larger trials.

## Methods

### Study design and setting

This prospective, outcome-assessor-blinded, parallel-group randomized controlled trial was conducted at the Fujian Provincial Urology Medical Center in China from March 2024 to February 2025. The first participant was enrolled on 29 March 2024. The study protocol was approved by the Human Research Ethics Committee of Fujian Provincial Hospital before participant enrolment (approval No. K2024-03–008) and registered with the Chinese Clinical Trial Registry (ChiCTR2400094812) on 27 December 2024. Because registration was completed after enrolment of the first participant, the trial was retrospectively registered.

The original ethics-approved protocol specified the randomized controlled design, intervention and control conditions, repeated perioperative assessment time points, the primary functional outcome domain including the 6-min walk test used for sample-size estimation, and the intention-to-treat principle before participant enrolment began. All participants provided written informed consent, and all procedures adhered to the ethical principles established by the Declaration of Helsinki and its subsequent revisions.

### Participants

Potential participants were consecutively screened at urology specialist outpatient clinics across both campuses of the centre. A trained research nurse reviewed clinic appointment lists daily and approached potentially eligible patients during outpatient visits.

Participants were eligible if they met all of the following criteria: (1) aged 60 years or older; (2) biopsy-confirmed prostate cancer and scheduled for elective radical prostatectomy; (3) an expected interval of more than one week between enrolment and surgery, allowing delivery of the short-term prehabilitation intervention; (4) prefrailty or frailty according to the Fried Frailty Phenotype [22], defined as one to two and three or more criteria, respectively; and (5) provision of written informed consent.

Participants were excluded if they met any of the following criteria: (1) multiple primary cancers or metastatic malignancies; (2) receipt of neoadjuvant radiotherapy, chemotherapy, androgen deprivation therapy, or other systemic anticancer treatment before enrolment; (3) mobility limitations or medical contraindications to exercise identified during standard preoperative assessment, defined as any unstable cardiovascular, respiratory, infectious, musculoskeletal, neurological, or other physician-determined condition that made preoperative exercise unsafe; or (4) inability to understand the study procedures, provide written informed consent, or complete the home-based intervention and repeated outcome assessments.

### Randomization and blinding

A computer-generated randomization sequence, created using IBM SPSS Statistics version 26.0, allocated participants in a 1:1 ratio to either the prehabilitation or usual care group. Allocation concealment was maintained through sequentially numbered, opaque, sealed envelopes. These envelopes were prepared by an independent staff member not involved in recruitment, intervention delivery, outcome assessment, or data analysis, and were opened sequentially only after baseline assessment had been completed.

Because of the behavioural nature of the intervention, participants and intervention providers could not be blinded to group allocation. However, outcome assessors remained blinded throughout data collection. For the primary statistical analysis, group labels were coded and masked until the main analyses were completed.

### Intervention and usual care

#### Short-term home-based multimodal prehabilitation programme

The short-term home-based multimodal prehabilitation programme was developed before trial initiation through literature review, expert consultation, pilot testing, and adaptation of a previously established lifestyle-integrated multicomponent exercise programme for frail older adults [23]. It was designed as a pragmatic, risk-targeted intervention for prefrail and frail older men awaiting radical prostatectomy within the limited and variable preoperative period.

The programme was delivered individually from enrolment until the day before surgery, using an initial face-to-face assessment and instruction followed by home-based practice and structured remote supervision, with actual duration determined by surgical scheduling.

The programme comprised three core components—exercise training, nutritional optimisation, and psychological support—along with three procedure-specific components—pelvic floor muscle training, respiratory exercises, and perioperative health behaviour guidance. Supplementary Fig. 1 illustrates the overall implementation pathway. The intervention is reported according to the SOS-Prehab framework in Supplementary Table 1 [24], including intended benefits, target behaviours, providers, setting, delivery mode, prescribed dose, tailoring rules, adherence assessment, and safety monitoring for each component.

At baseline, participants underwent an individualised assessment of frailty status, physical performance, exercise tolerance, nutritional risk, and psychological distress. The starting dose and support intensity were tailored according to this assessment. Standardised provider training, written materials, audio guidance, rehabilitation diaries, and predefined progression/regression rules were used to support intervention fidelity.

The exercise component embedded aerobic, resistance, and balance activities into daily routines, such as marching on the spot, figure-of-eight walking, arm curls, chair-sit-to-stand, with intensity guided by perceived exertion and symptom tolerance. Nutritional optimisation was guided primarily by the Nutritional Risk Screening 2002, dietary intake, and clinical status, with individualised dietary advice provided when indicated. Psychological support focused on emotional reassurance, coping guidance, and referral for individualised support when clinically indicated. Participants with a Hospital Anxiety and Depression Scale subscale score of 11 or higher were considered for additional psychological support. Procedure-specific components included pelvic floor muscle training to support postoperative urinary recovery, respiratory exercises to improve respiratory preparedness for early mobilisation, and perioperative health-behaviour guidance to reinforce self-management during the surgical waiting period.

During the home-based intervention period, participants recorded daily completion of intervention components, perceived exertion, symptoms, and reasons for non-completion in a daily prehabilitation diary. WeChat or text messaging was used for routine check-ins, diary submission, reminders, reinforcement, and brief feedback. Telephone calls were used when participants reported symptoms, required clarification of training techniques, experienced adherence difficulties, or preferred verbal communication. Specialist nurses conducted structured remote follow-up every 1–2 days, with additional contact when needed, to review diary records, assess tolerance, address barriers, provide motivational support, and adjust the plan according to predefined rules.

Programme execution rate was assessed using prehabilitation diaries and WeChat check-in records and was defined as the proportion of prescribed intervention days on which the multimodal prehabilitation programme was completed. Adverse events were defined as any new or worsening unintended medical occurrence during the intervention period, regardless of causality, including symptoms requiring programme modification, interruption, or discontinuation.

#### Usual care

Participants in the usual care group received standard perioperative management following the institutional Enhanced Recovery After Surgery (ERAS) protocol for radical prostatectomy. This included routine preoperative assessment, standard perioperative education, general advice on diet and physical activity, and usual surgical preparation. Before surgery, usual care did not include individualised exercise prescriptions, structured nutritional optimisation, psychological support programmes, pelvic floor muscle training programmes, structured respiratory exercise programmes, or remote adherence monitoring.

Postoperative care followed the same standardized ERAS pathway in both groups. Staff involved in postoperative mobilisation and discharge-related decisions were not formally blinded to group allocation; however, these decisions were guided by predefined institutional ERAS protocols and clinical criteria rather than study allocation.

### Outcome measures

Outcome assessments were conducted at four predefined time points: baseline before intervention (T1), the day before surgery (T2), the day of discharge (T3), and 4 weeks postoperatively (T4).

The primary outcome was functional capacity, assessed by the 6-min walk test (6MWT) across the perioperative period. The 6MWT was selected because it reflects submaximal functional exercise capacity and walking endurance relevant to daily activities in older surgical patients [25].

Supportive secondary outcomes included frailty-related physical performance measures. Frailty status was assessed using the Fried Frailty Phenotype (FFP) [26]. Physical performance was assessed using the 4-m gait speed test (4MGS) [27], bilateral handgrip strength (HGS) measured with a calibrated Jamar® hydraulic dynamometer [28], and the five-times sit-to-stand test (FTSST) [29].

Exploratory perioperative recovery outcomes included time to first ambulation, time to first flatus, drainage duration, length of postoperative hospital stay, urinary incontinence at 4 weeks [30], and total hospital costs. For this study, urinary incontinence was defined according to International Continence Society terminology as any involuntary leakage of urine.

Other exploratory outcomes included nutritional risk, serum albumin, psychological well-being, and health-related quality of life. Nutritional risk was assessed using the Nutritional Risk Screening 2002 (NRS-2002) [31]. Serum albumin [32] was collected as supplementary clinical information related to nutrition-inflammation status. Psychological well-being and health-related quality of life were assessed using HADS [33] and EORTC QLQ-C30 [34], respectively.

### Sample size calculation

Sample size was calculated using G*Power (version 3.1.9.7) based on the primary outcome, the 6-min walk test (6MWT). Based on data from a previous trial [35], a mean between-group difference of 72.4 m and a pooled standard deviation of 74.19 m were assumed. With a two-sided α of 0.05 and a power of 0.90, 23 participants were required per group. Accounting for a 20% attrition rate, the required sample size increased to 29 participants per group (58 in total). Given the uncertainty regarding attrition and the expected intervention effect in prefrail and frail older surgical patients, we conservatively increased the target sample size to 80 participants, with 40 participants in each group. The calculation was based on a conventional two-group comparison because reliable parameters for a GEE-specific calculation were unavailable in this population.

### Statistical analysis

Statistical analyses were performed using IBM SPSS Statistics (version 26.0) and followed the intention-to-treat principle. For participants who discontinued the intervention or did not complete follow-up assessments, missing repeated-measures outcome values were handled using the last observation carried forward method. For outcomes assessed only at 4 weeks postoperatively, observed denominators were reported, and dichotomous 4-week outcomes were not imputed.

Continuous variables were summarised as mean ± standard deviation (SD) for normally distributed data and as median (interquartile range, IQR) for non-normally distributed data. Categorical variables were summarised as frequencies and percentages. Baseline characteristics were compared between groups using the independent-samples t-test, Mann–Whitney U test, chi-square test, or Fisher’s exact test, as appropriate.

The primary outcome was functional capacity measured by the 6MWT across the perioperative period. The primary comparison was the between-group difference in change in 6MWT from baseline to the day before surgery. Repeated-measures outcomes were analysed using GEE, with group, time, and group *x* time interaction terms included in the model and an exchangeable working correlation structure was specified. The primary treatment effect was estimated from the group *x* time contrast for the baseline-to-preoperative change in 6MWT. GEE was used to account for within-participant correlation across repeated measurements and to estimate the population-average effect of the intervention on the perioperative 6MWT trajectory.

Supportive secondary repeated-measures outcomes, including frailty-related physical performance, and other exploratory repeated-measures outcomes, including nutritional status, psychological outcomes, and quality-of-life scores, were analysed using the same GEE framework. Exploratory perioperative recovery outcomes were analysed using Mann–Whitney U tests for continuous variables and chi-square or Fisher’s exact tests for categorical variables, as appropriate. No formal adjustment for multiple comparisons was applied. All tests were two-sided, and *P <* 0.05 was considered statistically significant.

## Results

### Participant flow, analysis population, and baseline characteristics

**Fig. 1:**
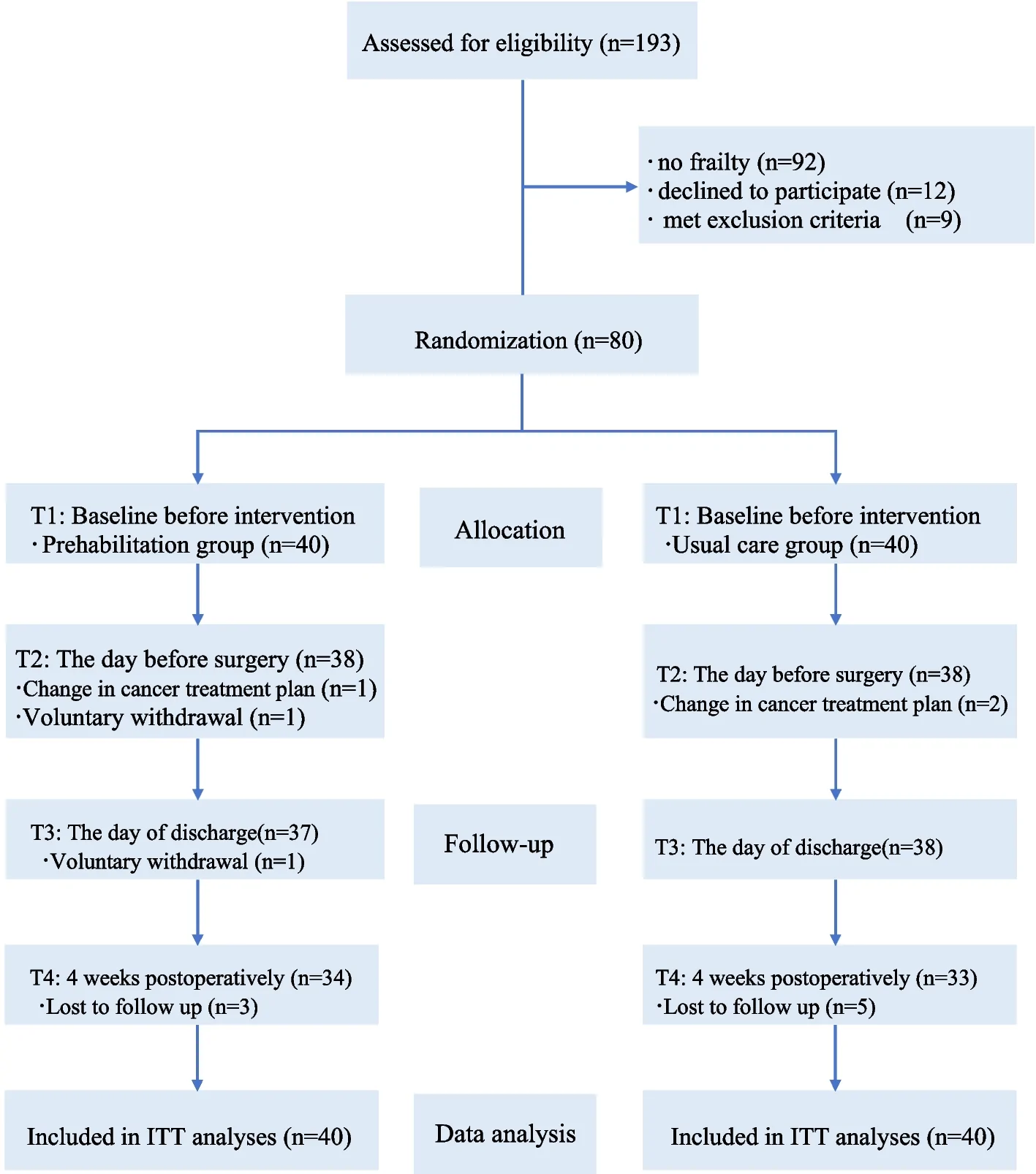
Participant flow diagram

A total of 193 patients with prostate cancer were assessed for eligibility. Of these, 113 were excluded: 92 did not meet the frailty eligibility criterion, 12 declined participation, and 9 met other exclusion criteria. The remaining 80 participants were randomized to the prehabilitation group (*n = *40) or the usual care group (*n = *40). The participant flow throughout the trial is presented in Fig. 1.

During the intervention and follow-up period, 6 participants in the prehabilitation group and 7 in the usual care group did not complete all scheduled follow-up assessments by 4 weeks postoperatively. All 80 randomized participants were included in the intention-to-treat analysis.

Baseline demographic and clinical characteristics were generally comparable between groups (Table 1). The median age of the total sample was 68.0 years, and 51 participants (63.8%) were classified as prefrail and 29 (36.2%) as frail at enrolment. Disease-related characteristics, including clinical stage, Gleason score, surgical approach, and ASA class, were comparable between groups.

**Table 1:** Baseline characteristics of the participants

Characteristics	Total (*n = *80)	Prehabilitation group (*n = *40)	Usual care group (*n = *40)	*t/Z/χ*^*2*^	*P* value
Age (years)	68.00 (63.25,73.75)^*^	68.00 (64.00,72.00)^*^	66.50 (63.00,67.00)^*^	−0.256^a^	0.798
BMI (Kg/m^2^)	24.54 ± 2.69	24.13 ± 2.74	24.95 ± 2.61	−1.382^b^	0.171
Education level (n, %)				0.099^d^	0.992
< Primary school	26 (32.5)	13 (32.5)	13 (32.5)		
Primary school	17 (21.3)	8 (20.0)	9 (22.5)		
Senior high school	25 (31.3)	13 (32.5)	12 (30.0)		
≥ College	12 (15.0)	6 (15.0)	6 (15.0)		
Household monthly income (CNY) (n, %)				2.412^c^	0.511
< 3000	22 (27.5)	12 (30.0)	10 (25.0)		
3000 ~ 6000	38 (47.5)	17 (42.5)	21 (52.5)		
6000 ~ 10,000	14 (17.5)	9 (22.5)	5 (12.5)		
> 10,000	6 (7.5)	2 (5.0)	4 (10.0)		
Smoking (n, %)				0.277^d^	0.871
Current	16 (20.0)	8 (20.0)	8 (20.0)		
Never	42 (52.5)	20 (50.0)	22 (55.0)		
Former	22 (27.5)	12 (30.0)	10 (25.0)		
Alcohol consumption (n, %)				0.577^c^	0.836
Current	8 (10.0)	5 (12.5)	3 (7.5)		
Never	52 (65.0)	25 (62.5)	27 (67.5)		
Former	20 (25.0)	10 (25.0)	10 (25.0)		
Comorbidities (n, %)				2.400^d^	0.121
None	20 (25.0)	13 (32.5)	7 (17.5)		
Present	60 (75.0)	27 (67.5)	33 (82.5)		
Polypharmacy (n, %)				0.891^d^	0.640
0	29 (36.3)	13 (32.5)	16 (40.0)		
1 ~ 2	30 (37.5)	17 (42.5)	13 (32.5)		
≥ 3	21 (26.3)	10 (25.0)	11 (27.5)		
Clinical stage (n, %)				2.998^c^	0.419
T1	21 (26.3)	11 (27.5)	10 (25.0)		
T2	48 (60.0)	25 (62.5)	23 (57.5)		
T3	6 (7.5)	1 (2.5)	5 (12.5)		
T4	5 (6.3)	3 (7.5)	2 (5.0)		
Gleason score (n, %)				4.073^c^	0.387
5	1 (1.3)	1 (2.5)	0 (0)		
6	40 (50.0)	23 (57.5)	17 (42.5)		
7	16 (20)	7 (17.5)	9 (22.5)		
8	13 (16.3)	4 (10.0)	9 (22.5)		
9	10 (12.5)	5 (12.5)	5 (12.5)		
Surgical approach (n, %)				0.054^d^	0.816
LRP	51 (63.8)	25 (62.5)	26 (65.0)		
RARP	29 (36.3)	15 (37.5)	14 (35.0)		
ASA (n, %)				1.656^c^	0.474
1	1 (1.3)	1 (2.5)	0 (0)		
2	25 (31.3)	14 (35.0)	11 (27.5)		
3	54 (67.5)	25 (62.5)	29 (72.5)		
Frailty (n, %)				0.216^d^	0.642
Prefrail	51 (63.8)	27 (67.5)	24 (60.0)		
Frail	29 (36.2)	13 (32.5)	16 (40.0)		

*ASA* American Society of Anesthesiologists

^a^Mann-Whitney U test;

^b^Independent samples t-test;

^c^Fisher exact test;

^d^Pearson ***x***^***2***^test

^*^Indicates non-normally distributed data, expressed as median (25th percentile, 75th percentile)

### Intervention exposure, programme execution rate, and safety

Among participants allocated to prehabilitation, the median intervention duration was 12 days (range 8–25 days). The mean overall programme execution rate was 79.3%. Mean component-specific completion rates were 87.0% for nutritional optimisation, 80.5% for pelvic floor muscle training, 78.2% for personalised exercise training, and 71.6% for respiratory exercises. No intervention-related adverse events were reported during the monitored prehabilitation period. No participant required programme modification, interruption, or discontinuation for safety reasons.

### Primary outcome: 6-min walk distance

**Fig. 2:**
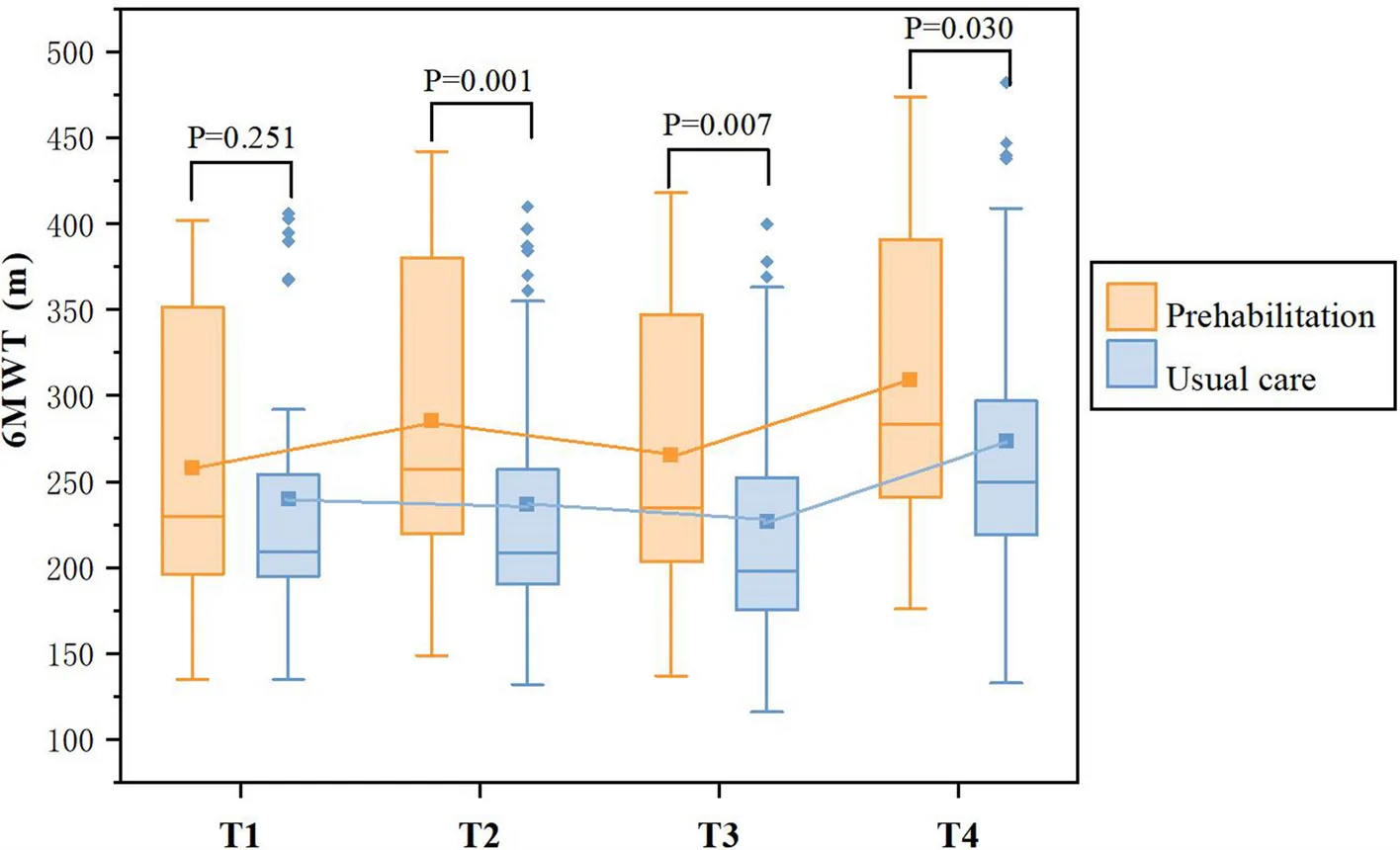
Perioperative trajectory of 6-min walk test distance. Measurements were made at four time points: T1, baseline; T2, the day before surgery; T3, discharge; T4, 4 weeks postoperatively. Box plots display the median (center line), interquartile range (box), and values within 1.5 times the interquartile range (whiskers); dots indicate outliers. Superimposed lines represent group mean values over time. *P* values indicate between-group comparisons at each time point

The perioperative distribution of 6MWT values is shown in Fig. 2, and between-group comparisons are presented in Table 2. Crude 6MWT values were comparable between groups at baseline. The primary comparison was the between-group difference in change in 6MWT from baseline to the day before surgery. The prehabilitation group improved by 27.70 ± 16.86 m, whereas the usual care group changed by − 3.12 ± 3.62 m, yielding a between-group difference of 30.82 m (95% CI, 25.32 to 36.33; *P <* 0.001).

**Table 2:** Primary outcome: 6-min walk distance

Outcome	Prehabilitation group (*n = *40)	Usual care Group (*n = *40)	Between-group difference (95% CI)	*P* value	GEE β (95% CI)	*P* value
6MWT, m—Baseline	230.0 (196.0, 343.8)	209.5 (195.0, 247.0)	20.5 (−20.0 to 44.0)	0.253	—	—
6MWT, m—Day before surgery	257.0 (219.8, 375.0)	208.5 (190.8, 250.0)	48.5 (13.5 to 75.5)	< 0.001	30.83 (25.55 to 36.10)	< 0.001
6MWT, m—Discharge	235.0 (204.2, 346.0)	198.0 (175.8, 245.8)	37.0 (10.0 to 70.0)	0.007	20.97 (14.72 to 27.23)	< 0.001
6MWT, m—4 weeks after surgery	283.5 (242.0, 390.5)	250.0 (220.0, 295.0)	33.5 (0.5 to 78.0)	0.030	17.50 (7.08 to 27.92)	0.001
Change in 6MWT from baseline to day before surgery, m	27.70 ± 16.86	−3.12 ± 3.62	30.82 (25.32 to 36.33)	< 0.001	30.83 (25.55 to 36.10)	< 0.001

Observed 6MWT values are presented as median (IQR); change scores are presented as mean ± SD. Between-group difference for observed values are descriptive median differences with bootstrap 95% CIs, and *P* values were calculated using Mann–Whitney U tests. The baseline-to-preoperative change was the primary comparison. The GEE model included group, time, and group*x*time interaction terms with an exchangeable working correlation structure

*GEE* Generalized estimating equations, *6MWT* 6-min walk test, *IQR* Interquartile range, *CI* Confidence interval

In the GEE analysis, the group *x* time contrast for the baseline-to-preoperative change in 6MWT was 30.83 m (95% CI, 25.55 to 36.10; *P <* 0.001), indicating a more favourable perioperative 6MWT trajectory in the prehabilitation group. Time point-specific comparisons at discharge and 4 weeks after surgery are reported descriptively in Table 2 to show the postoperative recovery trajectory.

### Supportive secondary outcomes: frailty-related physical performance

Supportive secondary outcomes related to frailty and physical performance are presented in Table 3, with longitudinal trajectories shown in Supplementary Fig. 2. Significant group *x* time interactions were observed for the FFP score, 4MGS, HGS, and the FTSST. Across follow-up, the prehabilitation group showed a generally more favourable pattern of frailty-related physical performance than the usual care group, including lower FFP score, shorter 4MGS and FTSST completion times, and higher HGS. For the FFP score, the between-group contrast was significant from baseline to the day before surgery and from baseline to 4 weeks postoperatively, but not from baseline to discharge.

**Table 3:** Supportive secondary outcomes: frailty-related physical performance

Outcomes	Overall group *x* time interaction *Wald χ*^*2*^ (*P*)	Contrast	GEE β (95% CI)	*P* value
FFP	25.825 (< 0.001)	Baseline to day before surgery	−0.85 (−1.21 to −0.49)	< 0.001
		Baseline to discharge	−0.38 (−0.86 to 0.11)	0.130
		Baseline to 4 weeks after surgery	−0.45 (−0.87 to −0.03)	0.036
4MGS, s	110.726 (< 0.001)	Baseline to day before surgery	−0.85 (−1.02 to −0.68)	< 0.001
		Baseline to discharge	−1.73 (−2.10 to −1.36)	< 0.001
		Baseline to 4 weeks after surgery	−1.62 (−1.96 to −1.28)	< 0.001
Left HGS, kg	87.693 (< 0.001)	Baseline to day before surgery	3.83 (2.97 to 4.69)	< 0.001
		Baseline to discharge	3.19 (2.21 to 4.17)	< 0.001
		Baseline to 4 weeks after surgery	3.77 (2.58 to 4.95)	< 0.001
Right HGS, kg	80.457 (< 0.001)	Baseline to day before surgery	4.21 (3.22 to 5.19)	< 0.001
		Baseline to discharge	3.70 (2.74 to 4.66)	< 0.001
		Baseline to 4 weeks after surgery	4.32 (3.10 to 5.54)	< 0.001
FTSST, s	91.189 (< 0.001)	Baseline to day before surgery	−2.31 (−2.79 to −1.83)	< 0.001
		Baseline to discharge	−2.41 (−3.08 to −1.73)	< 0.001
		Baseline to 4 weeks after surgery	−2.41 (−3.08 to −1.74)	< 0.001

The GEE models included group, time, and group *x* time interaction terms with an exchangeable working correlation structure. Negative β values indicate lower scores or shorter completion times in the prehabilitation group relative to usual care, whereas positive β values indicate higher handgrip strength

*FFP* Fried Frailty Phenotype score, *4MGS* 4-m gait performance, *Left HGS* Left handgrip strength, *Right HGS* Right handgrip strength, *FTSST* Five-times sit-to-stand test, *GEE* generalized estimating equations, *CI* confidence interval

### Exploratory perioperative recovery outcomes

Exploratory perioperative recovery outcomes are summarized in Table 4. Compared with usual care, the prehabilitation group showed signals consistent with faster early postoperative recovery, including shorter time to first ambulation (26.0 [23.0, 28.0] vs 29.0 [26.8, 32.0] h), shorter time to first flatus (32.0 [29.0, 34.0] vs 39.0 [36.0, 41.0] h), and shorter postoperative hospital stay (6.0 [5.0, 6.0] vs 7.0 [6.0, 9.5] d). Urinary incontinence at 4 weeks was observed in 13 of 34 participants (38.2%) in the prehabilitation group and 20 of 33 participants (60.6%) in the usual care group. Detailed between-group differences and 95% confidence intervals are provided in Table 4.

**Table 4:** Exploratory perioperative recovery outcomes

Outcome	Prehabilitation Group (*n = *40)	Usual care Group (*n = *40)	Between-group effect (95% CI)	*P* value
Time to first ambulation, h	26.0 (23.0, 28.0)	29.0 (26.8, 32.0)	−4.0 (−5.0 to −2.0)	< 0.001
Time to first flatus, h	32.0 (29.0, 34.0)	39.0 (36.0, 41.0)	−7.0 (−9.0 to −5.0)	< 0.001
Drainage duration, d	4.5 (3.7, 5.0)	5.0 (4.0, 6.1)	−0.5 (−1.2 to 0.0)	0.031
Postoperative hospital stay, d	6.0 (5.0, 6.0)	7.0 (6.0, 9.5)	−2.0 (−3.0 to −1.0)	< 0.001
Hospital cost, CNY	30,701 (27,657, 53,862)	36,727 (29,667, 55,290)	−2460 (−6052 to 612)	0.109
Urinary incontinence at 4 weeks, n/N (%)	13/34 (38.2%)	20/33 (60.6%)	−22.4 (−45.7 to 1.0)	0.089

Values for continuous outcomes are presented as median (IQR). Between-group effects for continuous outcomes are median differences with 95% CIs; binary outcomes are presented as risk differences in percentage points with 95% CIs

*P* values were calculated using Mann–Whitney U tests for continuous outcomes and Fisher's exact tests for binary outcomes

*CI* Confidence interval, *IQR* Interquartile range

### Other exploratory outcomes

Other exploratory repeated-measures outcomes are shown in Supplementary Table 2. Significant group *x* time interactions were observed for HADS anxiety and depression scores, with lower scores in the prehabilitation group mainly at the preoperative and discharge assessments. Significant group *x* time interactions were also observed for QLQ-C30 functional scales, symptom scales, and global health status, with generally more favourable short-term scores in the prehabilitation group. Serum albumin did not show a significant group *x* time interaction. Given their exploratory nature, these findings were interpreted as hypothesis-generating.

## Discussion

In this randomized controlled trial of prefrail and frail older men undergoing radical prostatectomy, a short-term home-based multimodal prehabilitation programme delivered within the actual preoperative waiting period improved the perioperative trajectory of functional capacity measured by the 6-min walk test. The between-group difference was already evident before surgery after a median intervention duration of only 12 days, suggesting that even a brief, pragmatic model may enhance early functional readiness in older surgical patients with compromised physiological reserve. These findings should be interpreted as evidence of short-term improvement in perioperative functional capacity rather than durable recovery or broader clinical effectiveness.

The 6MWT was selected as the primary outcome because it reflects submaximal functional capacity and walking endurance relevant to daily activities in older surgical patients [36, 37]. The primary finding was a more favourable perioperative trajectory of 6MWT performance in the prehabilitation group, suggesting improved functional readiness before surgery and a possible contribution to early postoperative recovery of functional capacity. This is consistent with previous prehabilitation studies showing improved 6MWT performance in frail or high-risk surgical oncology populations [38, 39], and extends this evidence to prefrail and frail older men undergoing radical prostatectomy within a short, real-world preoperative waiting period. Nevertheless, the clinical significance of the observed change requires caution. The primary between-group difference in change before surgery was approximately 30.8 m; although potentially relevant in older patients with limited physiological reserve, established 6MWT minimal important difference thresholds are not specific to this population [40, 41]. Thus, the finding should be interpreted as statistically significant and potentially clinically relevant, rather than definitive evidence of a clinically meaningful functional benefit.

The short-term improvement in 6MWT performance should be interpreted in the context of both the intervention window and the target population. A median prehabilitation period of 12 days is unlikely to produce substantial increases in muscle mass, but early functional adaptations may occur through improved neuromuscular activation, walking efficiency, respiratory control, exercise familiarity, and confidence in physical activity [42, 43]. This may be particularly plausible in a population consisting largely of prefrail patients, in whom physiological reserve is compromised but potentially modifiable [44, 45]. The lifestyle-integrated design may also have facilitated short-term effects by embedding aerobic, resistance, and balance activities into daily routines, thereby increasing the feasibility and repetition of therapeutic movement without requiring additional hospital visits [23]. Directionally consistent changes in FFP score, 4MGS, HGS, and FTSST were consistent with the interpretation that the primary 6MWT finding reflected short-term improvement in functional readiness, although these outcomes were considered supportive rather than confirmatory.

The exploratory perioperative recovery findings were directionally consistent with the primary functional outcome. Earlier ambulation, faster gastrointestinal recovery, earlier drainage removal, and shorter postoperative hospital stay may indicate that improved preoperative functional readiness was accompanied by more favourable early recovery processes, which is consistent with previous evidence suggesting that prehabilitation may support mobilisation and early postoperative recovery in older or high-risk surgical patients [46, 47]. However, the present trial was smaller, shorter, and powered for the primary 6MWT outcome rather than clinical endpoints. Although larger trials such as PREHAB have reported benefits of longer multimodal prehabilitation for postoperative complications in other surgical populations [48], our perioperative recovery outcomes should be interpreted as exploratory signals rather than definitive evidence of reduced postoperative morbidity.

Exploratory patient-reported outcomes suggested that prehabilitation’s benefits may extend beyond physical recovery to psychological adaptation. Reduced perioperative anxiety and depression, alongside improved quality-of-life domains, may reflect the combined effects of structured support, increased patient engagement, and enhanced confidence in recovery [42]. However, these findings should be interpreted cautiously, given the exploratory nature of these outcomes and the lack of participant blinding. In contrast, serum albumin did not differ between groups over time. This finding is not unexpected, as albumin is influenced by inflammation, illness severity, fluid balance, hepatic synthesis, and protein losses, and should not be interpreted as a stand-alone marker of nutritional status or short-term nutritional response [49, 50].

From a clinical perspective, these findings suggest that a brief, lifestyle-integrated, home-based multimodal prehabilitation programme may be feasible within the short and variable waiting period before radical prostatectomy. The high participation rate may reflect the low-burden design, integration into the existing surgical pathway, nurse-led remote support, and established patient-clinician trust within this single-centre setting. However, these observations should not be interpreted as evidence of implementation effectiveness or generalisability. Longer follow-up is needed to determine whether early gains in 6MWT translate into sustained functional independence, urinary continence recovery, quality-of-life benefits, or reduced postoperative morbidity.

### Strengths and limitations

This study possesses several strengths. It addressed a clinically significant yet underrepresented population: prefrail and frail older men scheduled for radical prostatectomy. The intervention’s delivery during the actual preoperative waiting period enhances the practical relevance of its findings. Methodological rigor, including a randomized design, allocation concealment, blinded outcome assessment, intention-to-treat analysis, and repeated perioperative assessments, strengthens the study’s internal validity. Furthermore, the multimodal, home-based intervention, supported by structured follow-up, improves its potential for translation into routine surgical care. Although GEE has not been routinely emphasized in previous prehabilitation trials, its use may be particularly valuable for evaluating repeated perioperative recovery trajectories.

Several limitations should be noted. First, the trial was retrospectively registered, although the original ethics-approved protocol was available and the prespecified, supportive, exploratory analyses were clarified in the revised manuscript. Second, blinding of participants, intervention providers, and postoperative care staff was not feasible, which may have introduced performance or decision-making bias despite blinded outcome assessment and standardized ERAS pathways. Third, this single-centre trial had a moderate sample size and was powered for the primary 6MWT outcome rather than complications, urinary continence or other clinical endpoints. Fourth, missing follow-up data may have introduced uncertainty; therefore, 4-week outcomes were reported using observed denominators and findings were interpreted cautiously. Finally, the 4-week follow-up and lack of multiplicity adjustment limit conclusions regarding sustained recovery and exploratory outcomes. Larger multicentre trials with longer follow-up are needed.

## Conclusions

In prefrail and frail older men undergoing radical prostatectomy, short-term, home-based multimodal prehabilitation delivered within the actual preoperative waiting period improved the perioperative trajectory of functional capacity measured by the 6MWT. Supportive findings in frailty-related physical performance and selected early recovery processes suggest potential benefits for early functional readiness, but should be interpreted as exploratory. Larger multicentre trials with longer follow-up are needed to confirm whether these early functional gains translate into sustained recovery and broader clinical benefits.

## Supplementary Information

Supplementary Material 1.

Supplementary Material 2.

Supplementary Material 3.

Supplementary Material 4.

## Data Availability

The datasets generated during and/or analyzed during the present study will be available from the corresponding author on reasonable request.

## Abbreviations

6MWT: 6-Minute walk test
ERAS: Enhanced Recovery After Surgery
EORTC QLQ-C30: European Organization for Research and Treatment of Cancer Quality of Life Questionnaire Core 30
FFP: Fried Frailty Phenotype
GEE: Generalised estimating equations
HADS: Hospital Anxiety and Depression Scale
PC: Prostate cancer
RP: Radical prostatectomy
