# Supplementary material for: Short-term home-based multimodal prehabilitation for perioperative functional recovery in prefrail and frail older men undergoing radical prostatectomy: a randomized controlled trial
Source: BMC Geriatr. 2026 Jul 24;26:1163. doi: 10.1186/s12877-026-08031-3 (PMC13573488; doi:10.1186/s12877-026-08031-3)
Supplement: Supplementary file 1 [file 12877_2026_8031_MOESM3_ESM.pdf]

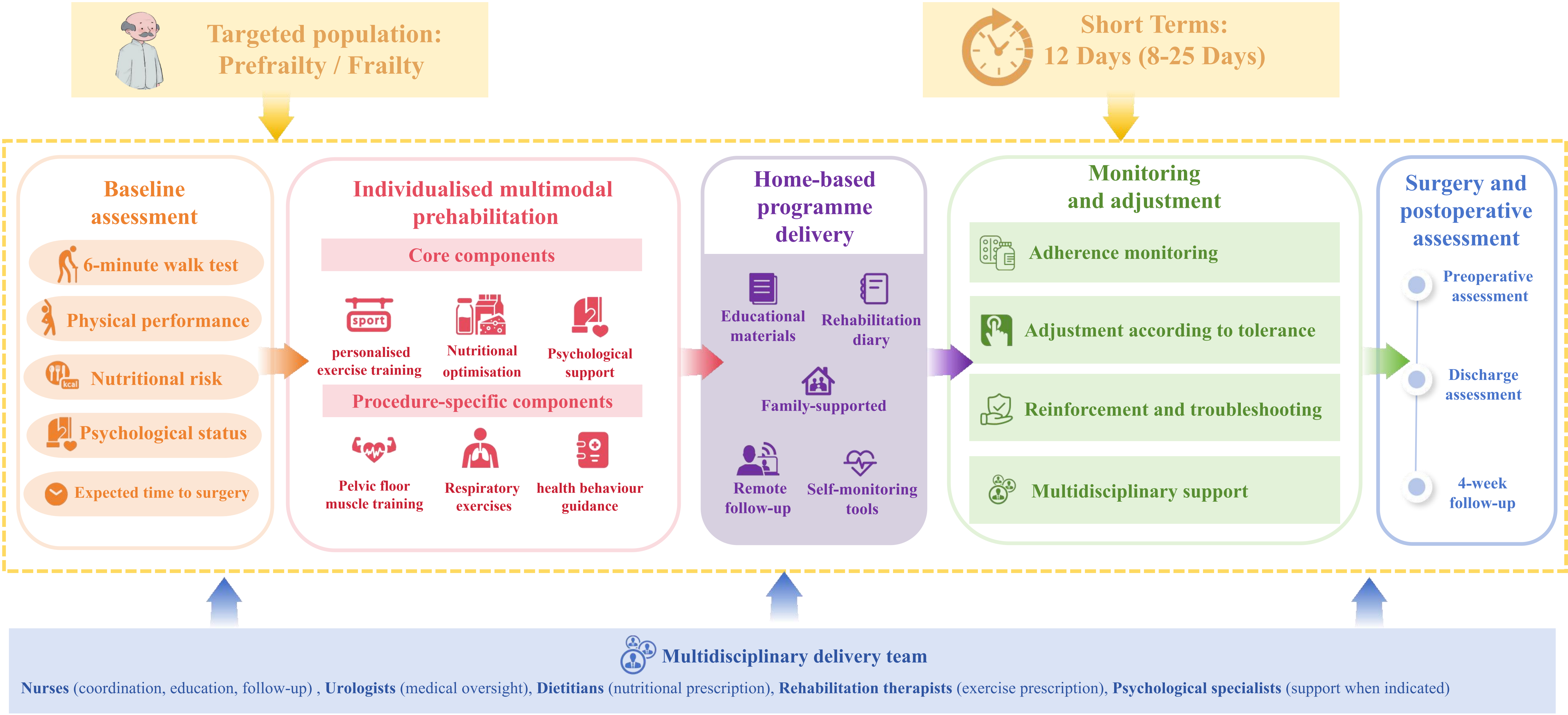

Supplementary Fig. 1 Implementation pathway of multimodal prehabilitation for radical prostatectomy
