# Supplementary material for: Short-term home-based multimodal prehabilitation for perioperative functional recovery in prefrail and frail older men undergoing radical prostatectomy: a randomized controlled trial
Source: BMC Geriatr. 2026 Jul 24;26:1163. doi: 10.1186/s12877-026-08031-3 (PMC13573488; doi:10.1186/s12877-026-08031-3)
Supplement: Supplementary file 2 [file 12877_2026_8031_MOESM4_ESM.pdf]

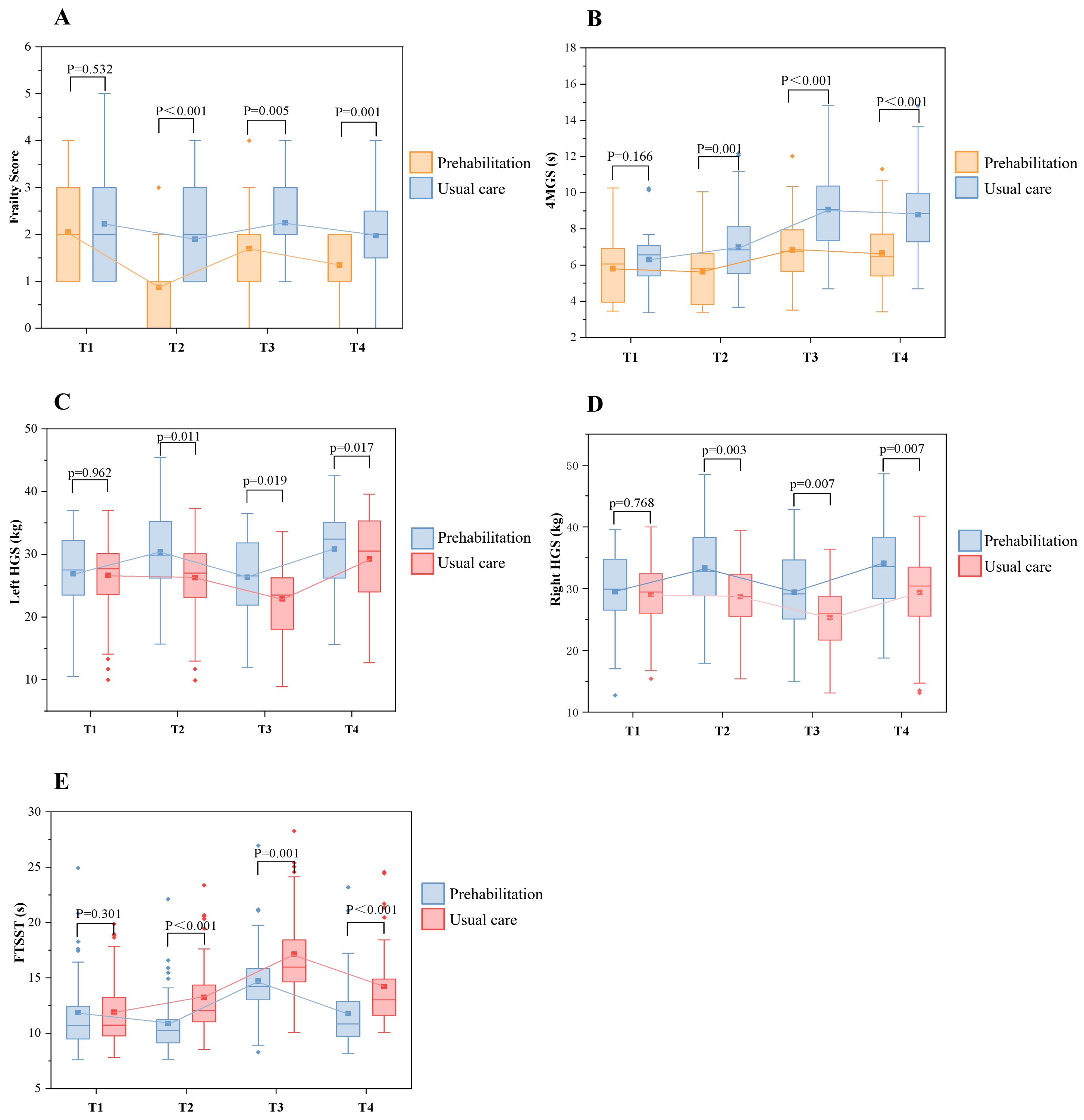

Supplementary Fig. 2 Trajectories of functional and frailty-related outcomes over time in the two groups.

(A) Fried Frailty Phenotype (FFP) score; (B)4-meter gait speed (4MGS); (C)handgrip strength of the left hand; (D)handgrip strength of the right hand; and (E)five-times sit-to-stand test (FTSST).

Measurements were made at four time points: T1, baseline; T2, the day before surgery; T3, discharge; T4, 4 weeks postoperatively.

Box plots display the median (center line), interquartile range (box), and values within 1.5 times the interquartile range (whiskers); dots indicate outliers. Superimposed lines represent group mean values over time.

P values indicate between-group comparisons at each time point.
