# Supplementary material for: Short-term home-based multimodal prehabilitation for perioperative functional recovery in prefrail and frail older men undergoing radical prostatectomy: a randomized controlled trial
Source: BMC Geriatr. 2026 Jul 24;26:1163. doi: 10.1186/s12877-026-08031-3 (PMC13573488; doi:10.1186/s12877-026-08031-3)
Supplement: Supplementary file 3 [file 12877_2026_8031_MOESM1_ESM.docx]

| **Supplementary Table 1.**  Multimodal prehabilitation programme reported according to the SOS-Prehab framework | | | | | | |
| --- | --- | --- | --- | --- | --- | --- |
| **Component** | **Intended benefit** | **Target behaviour / content** | **Provider, setting and mode** | **Prescribed dose** | **Tailoring / progression / regression** | **Monitoring, programme execution and safety** |
| Lifestyle-integrated multicomponent exercise | Improve functional capacity, mobility, balance, muscle strength and physical reserve. | Aerobic, resistance, and balance activities integrated into daily routines. Examples included marching on the spot, chair sit-to-stand, arm curls, supported single-leg stance, and figure-of-eight walking. The behavioural target was completion of the prescribed exercise programme on 5 non-consecutive days per week. | Initial face-to-face assessment and prescription by a rehabilitation therapist in the outpatient setting; home-based practice thereafter. Specialist nurses provided remote supervision and adjustment through WeChat or telephone every 1-2 days. | Twelve targeted exercises were prescribed, including four aerobic, four balance, and four resistance exercises. Each exercise was performed for 2–3 sets of 5–8 repetitions. | Exercise intensity was guided by the 0–10 rating of perceived exertion and symptom tolerance. If RPE was >7 or new/worsening symptoms occurred, exercise was stopped and the next-session dose was reduced by approximately 20%. If RPE was <3 and no symptoms occurred, the dose was increased by approximately 10%; otherwise, the dose was maintained. | Completion, RPE, symptoms and reasons for non-completion were recorded in rehabilitation diaries and reviewed during follow-up. Participants were instructed to stop activity and contact the research team if abnormal symptoms occurred; nurses actively screened for adverse events every 1-2 days. |
| Pelvic floor muscle training | Support pelvic floor function and early postoperative urinary recovery. | Kegel exercises and bridging movements. Participants were taught correct muscle identification, contraction and relaxation, and avoidance of compensatory abdominal or gluteal contraction. | Face-to-face nurse-led instruction in the outpatient setting, followed by home practice and remote reinforcement; urologist consultation was available when needed. | 5 sets/day, 10 repetitions/set. | The number of sets could be adjusted within 5-10 sets/day according to technique and tolerance. | Technique, completion and urinary symptoms were reviewed during follow-up and documented in the rehabilitation diary. Adverse symptoms prompted reassessment and, when necessary, urologist consultation. |
| Respiratory exercises | Improve respiratory function and preparedness for postoperative mobilisation. | Resisted diaphragmatic breathing, pursed-lip breathing and balloon-blowing exercises. | Nurse-led face-to-face instruction at initiation, followed by home practice and remote reinforcement. | Approximately 15 min/session, twice daily. | Correct technique was confirmed at initiation. Dose and technique were reinforced or modified according to tolerance and symptoms. | Adherence and tolerance were reviewed every 1-2 days using diaries and follow-up records. Dyspnoea, dizziness, chest discomfort or other abnormal symptoms triggered cessation and clinical assessment. |
| Nutritional optimisation | Support metabolic reserve and perioperative recovery. | Individualised dietary counselling and oral nutritional supplementation when clinically indicated. Nutritional risk was assessed primarily using NRS-2002 together with dietary intake and clinical status; serum albumin was supplementary information rather than a stand-alone nutritional marker. | Assessment and prescription by a clinical dietitian; nurses monitored intake, adherence and gastrointestinal tolerance through remote follow-up. | Energy target: approximately 30 kcal/kg/day; protein target: up to approximately 2.0 g/kg/day when clinically appropriate and not contraindicated. | Participants with NRS-2002 <3 received counselling and intake monitoring. Participants with NRS-2002 ≥3 underwent individualised dietitian assessment, and oral nutritional supplements were prescribed when clinically indicated. | Dietary intake, supplement use and gastrointestinal tolerance were reviewed during follow-up and documented. Intolerance prompted modification or dietitian reassessment. |
| Psychological support | Alleviate peroperative psychological distress and enhance coping. | Progressive muscle relaxation and mindfulness meditation using standardised audio recordings. | Standardised nurse-led support with home practice; referral to a clinical psychologist when indicated. | Approximately 20 min/day. | Participants with a HADS subscale score ≥11 were considered for referred or individualised psychological support when clinically indicated. Barriers to daily practice were addressed during follow-up. | Practice completion and psychological concerns were reviewed every 1-2 days. Any worsening distress or safety concern prompted referral for professional assessment. |
| Health behaviour guidance | Promote perioperative self-management and adherence. | Smoking cessation, alcohol reduction or avoidance, perioperative preparation education and reinforcement of programme goals. | Individual nurse-led counselling at the outpatient baseline visit, with reinforcement through WeChat or telephone follow-up. | Brief counselling at baseline with reinforcement throughout the preoperative period. | Behavioural goals were tailored to smoking and alcohol status, readiness to change, and reported barriers. | Progress and barriers were reviewed during follow-up contacts. Motivational interviewing and family support were used when appropriate. |
| Programme-level delivery and fidelity | Ensure consistent, reproducible and safe delivery of the multimodal programme. | Initial risk and needs assessment included frailty status, physical performance, exercise tolerance, nutritional risk, psychological distress, comorbidities, symptoms and contraindications. | The programme began after outpatient enrolment and continued until the day before surgery. Delivery consisted of an initial face-to-face session followed by home practice and structured remote supervision. No additional hospital visits were required solely for the intervention. | Intervention duration depended on the scheduled operation date. | Starting dose and support were individualised from baseline assessment. Standardised provider training, written materials, audio guidance, diaries and predefined adjustment rules were used to support intervention fidelity. | Specialist nurses reviewed diaries and daily check-ins every 1-2 days. A study-specific adverse-event form was available to record event description, management and outcome. Overall programme execution rate were reported. |
| Usual care comparator | Provide standard perioperative management without the structured multimodal prehabilitation package. | Routine preoperative assessment, general perioperative education, usual surgical preparation, and general advice regarding diet and physical activity. | Delivered by the usual clinical team according to the institutional pathway. | As scheduled within routine care. | No individualised multicomponent exercise prescription, structured progression/regression criteria, dietitian-directed optimisation, standardised relaxation practice, rehabilitation diary, or remote adherence monitoring before surgery. | Both groups received the same postoperative ERAS pathway. |
| **Abbreviations:** RPE, rating of perceived exertion; NRS-2002, Nutritional Risk Screening 2002; HADS, Hospital Anxiety and Depression Scale; ERAS, Enhanced Recovery After Surgery. | | | | | | |
