# Supplementary material for: Short-term home-based multimodal prehabilitation for perioperative functional recovery in prefrail and frail older men undergoing radical prostatectomy: a randomized controlled trial
Source: BMC Geriatr. 2026 Jul 24;26:1163. doi: 10.1186/s12877-026-08031-3 (PMC13573488; doi:10.1186/s12877-026-08031-3)
Supplement: Supplementary file 4 [file 12877_2026_8031_MOESM2_ESM.docx]

| **Supplementary Table 2**  Other exploratory repeated-measures outcomes from T1 to T4. | | | | | | | | |
| --- | --- | --- | --- | --- | --- | --- | --- | --- |
| **Variables** | **Groups** | **Time Point** | | | | Between  group effect  *Wald χ^2^* (*P*) | Within  group effect  *Wald χ^2^* (*P*) | Interaction effect  *Wald χ^2^* (*P*) |
|  |  | Baseline before intervention (T1) | The day before surgery (T2) | The day of discharge (T3) | 4 weeks postoperatively (T4) |  |  |  |
| **Albumin** | Prehabilitation group | 44.00 (42.00, 47.00)* | 44.00 (42.00, 46.00)* | 35.00 (33.00, 38.00)* | 41.00 (40.00, 42.00)* | 0.022  (0.882) | 352.602  (<0.001) | 5.316  (0.150) |
|  | Usual care group | 45.00 (42.00, 48.00)* | 45.00 (41.00, 47.00)* | 35.00 (33.00, 36.75)* | 41.00 (39.00, 42.00)* |  |  |  |
|  | *Z* | -1.035 | -0.993 | -1.330 | -0.656 |  |  |  |
|  | *P* | 0.301 | 0.321 | 0.184 | 0.512 |  |  |  |
| **HADS-A** | Prehabilitation group | 10.00 (8.00, 10.00)* | 8.00 (7.00, 9.00)* | 6.00 (5.00, 7.00)* | 6.00 (5.00, 6.00)* | 12.321  (<0.001) | 307.984  (<0.001) | 37.862  (<0.001) |
|  | Usual care group | 10.00 (8.00, 10.00)* | 10.50 (8.25, 12.00)* | 8.00 (7.00, 9.00)* | 6.00 (5.00, 7.00)* |  |  |  |
|  | *Z* | -0.040 | -4.241 | -4.988 | -1.669 |  |  |  |
|  | *P* | 0.968 | ＜0.001 | ＜0.001 | 0.095 |  |  |  |
| **HADS-D** | Prehabilitation group | 8.95±2.00 | 8.00 (7.00, 9.00)* | 7.00 (6.00,7.00)* | 7.00 (6.00, 7.00)* | 10.840  (0.001) | 112.440  (<0.001) | 45.929  (v0.001) |
|  | Usual care group | 9.00 (7.00, 11.00)* | 9.00 (7.25, 11.00)* | 8.95±1.87 | 7.10±1.72 |  |  |  |
|  | *t/Z* | -0.127 | -2.958 | -5.221 | -0.953 |  |  |  |
|  | *P* | 0.899 | ＜0.001 | ＜0.001 | 0.340 |  |  |  |
| **QLQ-C30-**  **Functional Scales** | Prehabilitation group | 426.67 (405.83, 453.33)* | 448.00±24.31 | 427.33±30.12 | 467.50 (453.33, 476.67)* | 18.245  (<0.001) | 201.589  (<0.001) | 44.773  (<0.001) |
|  | Usual care group | 426.38±41.08 | 408.21±47.37 | 370.37±62.64 | 445.84 (422.50, 464.17)* |  |  |  |
|  | *t/Z* | -1.425 | 4.727 | 5.183 | -3.395 |  |  |  |
|  | *P* | 0.154 | ＜0.001 | ＜0.001 | 0.001 |  |  |  |

| **Supplementary Table 2**  Other exploratory repeated-measures outcomes from T1 to T4 (continued). | | | | | | | | |
| --- | --- | --- | --- | --- | --- | --- | --- | --- |
| **Variables** | **Groups** | **Time Point** | | | | Between  group effect  *Wald χ^2^* (*P*) | Within  group effect  *Wald χ^2^* (*P*) | Interaction effect  *Wald χ^2^* (*P*) |
|  |  | Baseline before intervention (T1) | The day before surgery (T2) | The day of discharge (T3) | 4 weeks postoperatively (T4) |  |  |  |
| **QLQ-C30**  **Symptom Scales** | Prehabilitation group | 40.88±5.43 | 27.78 (2.78, 44.44)* | 36.11 (18.06, 65.28)* | 16.67 (0, 37.50)* | 10.071  (0.002) | 131.882  (<0.001) | 42.779  (v0.001) |
|  | Usual care group | 38.78 (22.22, 50.00)* | 38.89 (27.78, 59.72)* | 65.14±28.38 | 27.78 (22.22, 44.44)* |  |  |  |
|  | *t/Z* | 0.300 | -2.963 | -3.993 | -2.748 |  |  |  |
|  | *P* | 0.764 | 0.003 | ＜0.001 | 0.006 |  |  |  |
| **QLQ-C30**  **Global health status** | Prehabilitation group | 50.00 (41.67, 58.33)* | 58.33 (50.00, 58.33)* | 41.67 (33.33, 50.00)* | 66.67 (58.33,75.00)* | 3.888  (0.049) | 969.222  (<0.001) | 22.834  (<0.001) |
|  | Usual care group | 58.33 (50.00, 64.58)* | 50.00 (41.67, 58.33)* | 33.33 (25.00, 41.67)* | 58.33 (50.00, 66.67)* |  |  |  |
|  | *Z* | -1.263 | -2.802 | -3.438 | -1.828 |  |  |  |
|  | *P* | 0.207 | 0.005 | 0.001 | 0.068 |  |  |  |
| **Note:** HADS-A, Hospital Anxiety and Depression Scale—Anxiety; HADS-D, Hospital Anxiety and Depression Scale-Depression; QLQ-C30, European Organization for Research and Treatment of Cancer Quality of life Questionnaire.  * indicates non-normally distributed data, expressed as median (25th percentile, 75th percentile). | | | | | | | | |
